# Undiagnosed status and associated factors of hypertension among adults living in rural of central, Ethiopia, 2020: Uncovering the hidden magnitude of hypertension

**DOI:** 10.1371/journal.pone.0277709

**Published:** 2022-12-15

**Authors:** Firaol Regea Gelassa, Adamu Birhanu, Abera Shibiru, Shalama Lekasa Nagari, Desalegn Emana Jabena

**Affiliations:** 1 Assosa University, Colleges of Health sciences, Assosa, Ethiopia; 2 Ambo University, College of Medicine and Health sciences, Ambo, Ethiopia; University of Liverpool, UNITED KINGDOM

## Abstract

**Background:**

Hypertension is a major risk factor for cardiovascular (CVD) disease related deaths worldwide. It affects more than 20% of adults in Ethiopia, making it a major public health concern. Although it is important to uncover the hidden extent of hypertension, there is limited information on the proportion of undiagnosed hypertension in rural areas of the country.

**Objective:**

This study aimed to determine the magnitude of undiagnosed hypertension and associated factors among adults living in the rural Dano district, Central Ethiopia 2020.

**Methods and materials:**

A quantitative, community-based cross-sectional study conducted from May to July 2020. A three-stage sampling technique was used to select a total of 605 study participants. A Validated tool was used to assess the participant’s behavioral characteristics. Blood pressure was measured using digital blood pressure apparatus. The mean of three blood measurements was used to classify hypertension after intra-class correlation was tested. Standardized instruments were used to assess participants’ health-seeking behavior and knowledge of the hypertensive disease. The proportion of undiagnosed hypertension was determined among patients with hypertension. The regression analyses were done to determine factors associated with undiagnosed hypertension. The adjusted odds ratio with 95% CI was estimated to measure the strength of the association. The level of statistical significance was set at a p-value < 0.05.

**Results:**

The prevalence of undiagnosed hypertension was 21.32% (CI: 19.95%, 25.8%). Living in a household with the low wealth index [(AOR: 3.5,95%CI: (1.6,6.4)], far distance to health facility, [(AOR: 0.155,95%CI: (0.11.0.67)], underweight, [AOR = 2.2.1,95%CI:(2.00,4.22)], use of smokeless tobacco products, [AOR = 3.2,95%Cl:(1.88,4.75)], and participants’ knowledge of hypertension were independently associated with undiagnosed hypertension.

**Conclusion:**

This study shows that undiagnosed hypertension is a major public health problem in the study area. Living in a household with a low wealth index, being far from a health facility, being underweight, using smokeless tobacco products, and having little knowledge about hypertension increase the likelihood of having undiagnosed Hypertension. Hypertension health information, particularly to smokes tobacco users, could improve the perceived susceptibility to hypertensive disease, and reduce the hidden extent of hypertension.

## Introduction

Non-communicable diseases are a significant cause of death and disability worldwide in general and in low-and middle-income countries in particular [1]. Hypertension is defined as systolic blood pressure > 140 mm Hg and/or diastolic blood pressure > 90 mmHg [2]. Hypertension is the leading cause of cardiovascular-related death and disability world-wide [3]. Globally, more than 1.13 billion people reported having hypertension, 66.6% of whom live in Low income countries [4]. It accounted for about 37.5% of the disease burden, 45 of cardiovascular related death, 51% of stroke related death, 43.5% of all deaths and 3.7% of total Disability Adjusted Life Years (DALYs) [1,5]. Hypertension related direct medical cost is about 370 billion US$ per year world-wide and had had the effective management, more than 270 US$ per year could be profited [4,6,7].

Based on Ethiopia WHO STEP report, the national prevalence of High blood pressure in the adult population is 16% [8]. The pooled prevalence of hypertension in rural settings of the country was 18.5%. In some rural areas of the country, the prevalence of hypertension can reach up to 25% [9,10]. Hypertension complications are now the leading cause of hospitalization and death in the Ethiopia. According to a study, in Addis Ababa, Ethiopia, 11.3% of all medical admissions accounted for the complication of Hypertension and 14.6% of all medical ward deaths [10]. Another study conducted at Mekelle Hospital revealed that hypertension is responsible for 66.2% of Stroke cases [11].

Hypertension rarely causes symptoms and for many people goes undiagnosed for years until a serious medical problem occurs [12]. The symptoms that may be associated with hypertension are insignificant and may not serve to diagnose hypertension and make it unnoticeable [12]. Hypertension damages blood vessels and organ function, which increases the risk of developing several fatal complications, including heart attack, stroke, kidney failure, coronary artery disease, cerebrovascular accidents, and heart failure [13].

According to a 2016 WHO report, seven percent (7%) of global Cardiovascular diseases including Hypertension are delayed in screening and diagnosis which is caused by limited access to health care services due to lack of money, health information, remoteness, illiteracy, travel constraints and a limited number of health care facilities [14].

Early detection of hypertension is a critical first step action in hypertension care toward increasing hypertension care [15], preventing the disease’s complications [16], and saving a life [17]. Individuals with hypertension diagnosed early will benefit from a healthier lifestyle, better treatment, effective blood pressure control [17,18], and a lower risk of coronary heart disease and stroke [19]. However, because of its nature of silence, it may go undiagnosed until serious complications occur [12]. Ethiopia, WHO NCD STEP report 2015/2016, showed that the majority (79.9%) of the rural Ethiopian population had never been screened for their blood pressure [8].

Despite the importance of uncovering the hidden extent of hypertension, there was limited information on undiagnosed hypertension among adults living in rural areas in Ethiopia in general and in the study area particular. Previous studies [20,21] on undiagnosed hypertension focused on urban areas and addressed covariates of undiagnosed hypertension without considering social factors and some personal factors, which are classified as remote and immediate risk factors. Considering these factors, the prevalence and factors associated with undiagnosed hypertension were investigated in the present study.

## Materials and methods

**Fig 1:**
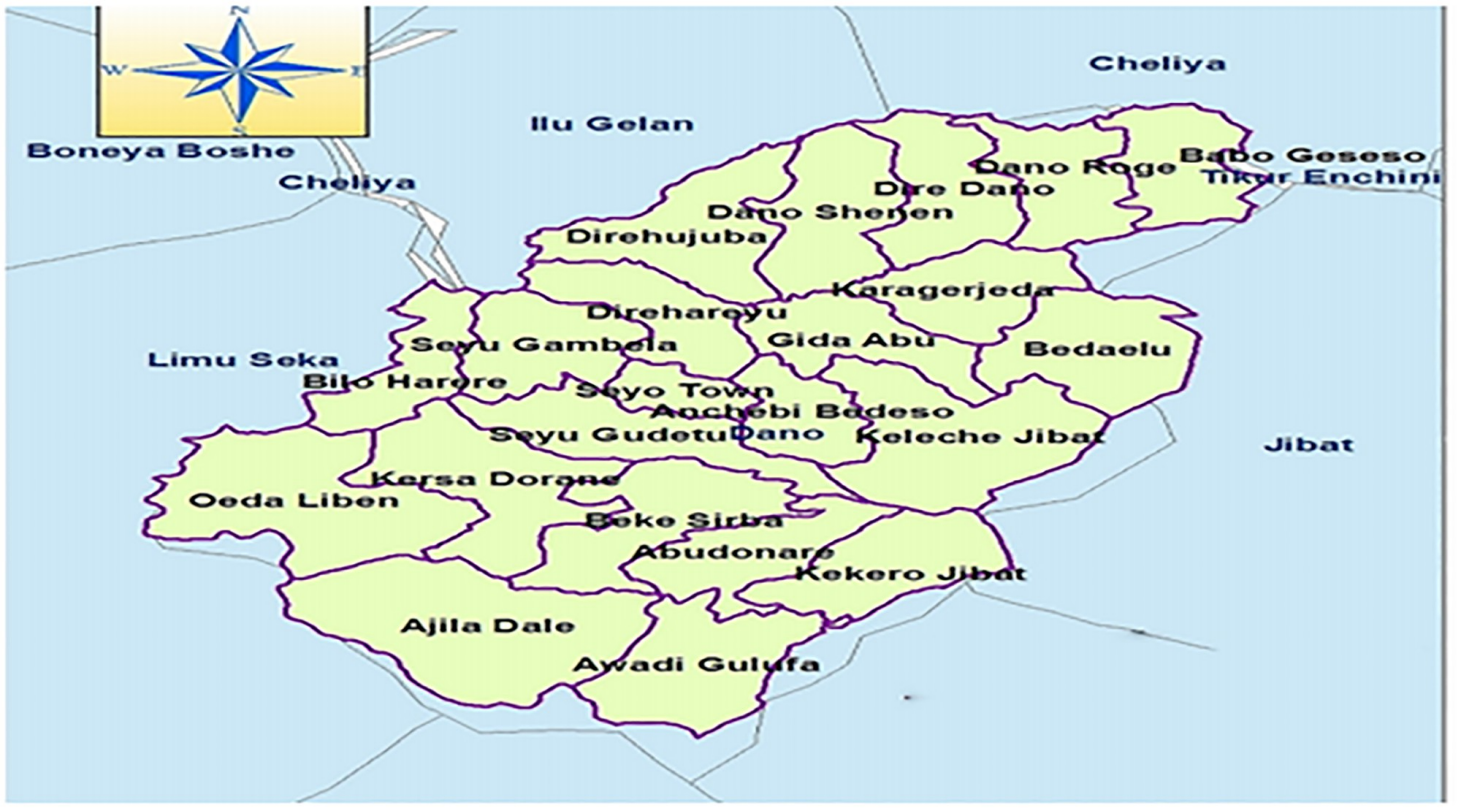
Map of Dano district to assess the prevalence of undiagnosed hypertension and associated factors among adults in 2020.

A quantitative community- based cross-sectional study was conducted with 605 study participants from Dano district. Dano district is located 225 kilometers west of Addis Ababa, the capital of Ethiopia, and Seyo is its largest city. The total population of Dano District is 122,618, of which 60,706 are men and 61,912 are women. Dano District has 27 Gandas (the small administrative unit in Oromia, Ethiopia), five urban administrative units, and 23 rural Gandas [22] (Fig 1).

### Populations

#### Source population

- All adults aged 19 to 65 years those who are living in the rural Gandas of the Dano district.

#### Study population

- The study population was all adults aged 19 to 65 years, living in randomly selected rural Gandas of the Dano district during the study period

#### Eligibility criteria

- **Inclusion Criteria—**those adults aged 19 to 65 years who are previously not diagnosed and not using anti-hypertensive drug.
- **Exclusion criteria—**Pregnant women’s and women’s’ in the post-partum period

### Sample size determination and sampling technique

#### Sample size determination

Sample size was determined by considering factors that are significantly associated with the outcome variable, two-sided confidence level of 95%, power 80% and the ratio of exposed to unexposed 1:1 for each factors, using Epi Info Version 7.2.2.6 (Table 1).

**Table 1:** Sample size for a factor associated with undiagnosed hypertension among adults living in the rural area of Dano district, Ethiopia 2020.

S/No	Factors	Reference	Assumptions	Sample size	10% non-response rate
1	Family history of hypertension AOR = 3.69(1.31,10)	9	Proportion exposed (having family history) = 26.8%	202	222
			Proportion in unexposed (Having no family history) = 10.5%		
2	Physical activity AOR 3.21(1.50,6.84)	9	Proportion exposed (physically in active) = 19.6%	292	321
			Proportion not exposed (physically active) = 7.7%		
3	Consume more salty food AOR = 3.67(1.210)	9	Proportion exposed (consume more salt) = 14.6%	366	403
			Proportion not exposed (Not consume less salt) 5.3%		
4	BMI AOR = 3.06(1.41–6.65)	9	Proportion exposed (Overweight) = 23.8%	222	244
			Proportion not exposed(normal weight) = 9%		

Then, the largest sample size (403) was considered for this study. Since the study was multi-stage, 1.5-design effect was considered and the final sample size became **605**.

#### Sampling procedure

In the Dano district, there are 23 rural Gandas (the smallest administrative unit in Oromia, Ethiopia). A multi-stage sampling technique was used to select the study participants. Stage one; seven rural Gandas were randomly selected by the lottery method. Stage two, selecting eligible individuals (i.e. not ever diagnosed with hypertension and/or not taking any hypertensive drug, non- pregnant mothers, and women’s, who are not in their post-partum period). Eligible individuals were enumerated depending on their legal residence and registered in prepared registration books to get a complete frame of the study population among randomly selected Gandas. In order to get those eligible individuals during data collection easily, the corresponding individual household was coded and registered in lining with their name. Stage three, the total sample size was allocated by probability proportional to size to each Gandas based on the number of eligible individuals they have as follow:

$$\text{ni}=\frac{\text{n}\times \text{Ni}}{\text{N}}$$

Where;

ni = the sample size of the i^th^ Ganda

Ni = population size of the i^th^ Ganda

n = n1+n2+n3+n4….n7 is the total sample size (605)

N = N1+N2+…N^th^—is total population size of those selected Gandas (11,067)

Finally, Six hundred five (605) participants were enrolled in the study using simple random sampling

### Data collection tools and techniques

Participants information on Socio-demographic variables behavioral and life style, physical measurement and biochemical measurement (blood glucose level) was collected using standardized WHO STEPS wise approach V.3.2 [23] tool that designed for surveillance of non-communicable disease. Participants health seeking behavior was assessed using questionnaire adopted from health belief model [24,25] and participants knowledge was assessed using a questionnaire which was adapted from Hypertension Knowledge-Level Scale (HK-LS) after reliability assessed for reliability [26].

The questionnaire that was developed in the English language was translated to Afan Oromo and return back to the English language to see for the consistency of both the English and Afan Oromo version of the questionnaire.

First, eligible adults were counted; the corresponding households were coded, and the individual’s name was tagged and recorded by health workers. Second, data were collected by health care professionals with at least a BSC degree in nursing and public health officials after they had received intensive training on the study objectives, survey procedures, and instruments from STEPS. Prior to the actual data collection, a pretest of the structured questionnaire was conducted in neighboring Ganda, which was not included in the study, to check the validity of the instruments and subsequently make any necessary corrections. Blood pressure was measured using, Riester ri-champion^®^N, automated and clinically validated (to the British Society for hematology standard) blood pressure measuring device with cuff size 22*43 width and length respectively. Participants were asked to rest (relax) in the chair for 30 minutes before measurement, not to cross their legs, to place their feet on the floor, to support their back and assure an empty bladder, not to smoke, and not to drink coffee/tea. The equipment was checked for integrity and disinfected with 70% isopropyl alcohol to protect it from COVID -19. Participants were then positioned with the arm supported on the desk to keep the upper arm level with the heart. The upper arm was exposed 2 inches above the crease of the elbow. The cuff was applied to the brachial artery on the upper arm. Three blood pressure measurements were taken 3 minutes apart in a sitting position. Finally, the mean of the three readings was taken to determine the BP status of the respondents.

Anthropometric measurements were performed using standard procedures and calibrated instruments. Participants’ weight was recorded to the nearest 0.1 kg. The height of the participants was measured using a portable stadiometer. It was recorded to the nearest 0.1 cm. Participants’ random and/or fasting blood glucose was measured using the program/service.

### Operational definition

**Chronic Disease**–was defined if participants were having at least one of the following chronic disease (i.e. Diabetic Mellitus, Cardio vascular disease, Cancer, COPD and Chronic kidney disease

**Screened status**—Participants said screened for the given chronic disease (Hypertension and DM) if he/she ever checked for chronic disease; otherwise, unscreened [27]

**Undiagnosed–**The participant is said undiagnosed, if reported having checked for chronic disease ever but not reported prior diagnosis [27]

**Family wealthy index–**is an index of the economic status of the household. Households of the participants will be ranked into quintiles as poor, middle and wealthy group depending on the house hold wealthy indices.

**Health seeking behavior**—was defined as a participant having a score of ≥ to the mean of each of the target dimensions was equated with having a high level of health-seeking behavior to hypertension, whereas participant having a score below the mean on each of the target dimensions was equated with having a low level of health-seeking behavior to hypertension

**Level of knowledge—**Good knowledge is if the Participants total knowledge score is above the mean score of the total knowledge question, while low knowledge is if the participant’s total knowledge score is equal or below the mean score of the total knowledge question.

### Data processing and analysis

The completed questionnaires were coded and entered into the computer program Epi Data version 3.1. SPSS version 25.0 was used for the analysis. Data were cleaned and edited using simple frequencies and cross tabulations before analysis. The cleaned final data were then analyzed using SPSS version 25.0.

In the analysis of outcome variables, the intra-class correlation coefficient (ICC) was tested to determine within-observation reliability for both systolic and diastolic blood pressure. Subsequently, the mean of three blood pressure measurements was used to classify the participants’ blood pressure. Accordingly, the mean systolic blood pressure > 140 mmHg and/or diastolic blood pressure > 90mmHg was coded as 1, and the mean systolic blood pressure < 140 mmHg and/or diastolic blood pressure < 90 mmHg was coded as 0.1.

The participant’s BMI was calculated from the person’s height and weight.

Participants’ knowledge of hypertension was analyzed from data collected using a knowledge questionnaire. For this purpose, the total knowledge score was calculated from 20 questions designed to measure participants’ knowledge about causes, risk factors, and prevention methods of hypertension. Adults with a score of 23.3 or more were coded as zero, and those with a score less than 23.3 were coded as one. Participants’ history of chronic disease was coded 1 if the answer was “yes” and 0 if the answer was “no.”

Bi-variable logistic regression analyzes were performed to determine the association between each independent variable and the outcome variable. Although the variables with a p-value < of 0.20 in the bi-variable logistic regression analysis were a candidate for the multivariable logistic regression analysis, they were ranked based on their p-value to be included in the multivariable logistic regression analysis because there were more variables than should be included in the multivariable logistic regression. The fit of the logistic regression model was checked using Hosmer-Lemeshow and statistics. Multicollinearity was checked (VIF < 10), indicating that there was no multicollinearity among the variables in this study. Both the crude and adjusted odds ratios were estimated along with the 95% CI to measure the strength of the association. The level of statistical significance was set at a p value of less than 0.05. Tables and figures were used to present the results.

### Ethical approval and consent to participate

The study was approved by the Ambo University, Medicine, and Health Sciences College Health Research Ethics Review Committee (CHRERC). The permission and support letter was obtained from the Dano district health department. Voluntary informed, written, and signed consent was obtained from all subjects after describing the nature and purpose of the study by the language they can understand. Each enumerators and data collector oriented to follow the national COVID-19 protocol during enumeration and data collection period respectively. Each participant washes his or her hand before physical, biochemical measurement and Blood pressure measuring devices were disinfected using 70% Isopropyl Alcohol to protect them from COVID-19. Those participants, who identified with hypertension, and diabetic mellitus were given advice and linked to the nearby health institution for further investigation and treatment. Health education was given for pre-hypertensive participants.

## Results

### Socio-demographic, personal and, psychosocial characteristics

Of the 569 study participants, more than 307 (54%) were male. The meanage (±SD) of participants was 37.2 (± 11.85) years, and most of the 453 (79.6%) study participants were married. One in three 212(37.3%) participants did not attend formal education and more than half 349(61.3%) of the participants were farmers. Two out of five 227(39.9%) of the participants belonged to the first poorest quintile of the Household Wealthy Index (Table 2).

**Table 2:** Socio-demographic and personal and health-related characteristics of Adults living in the rural area of Dano district, Oromia, Ethiopia, 2020 (n = 569).

Variable	Category	Frequency	Percentage
Sex the participant’s	Male	307	54
	Female	262	46
Age of the participant’s	19- 33years	266	46.7
	34–48 years	192	33.7
	49–65 years	111	19.5
Participant’s religion status	Protestant	246	43.2
	Orthodox	169	29.7
	Muslim	135	23.7
	Waaqeffataa	19	3.3
Participant’s ethnicity	Oromo	535	94
	Amhara	34	6
Participants marital status	Married	453	79.6
	Never married	51	9.0
	Divorced/separated	29	5.1
	Widowed	36	6.3
Participant’s educational level	No formal education	212	37.3
	Grade 1–4	58	10.2
	Grade 5–8	146	25.7
	Grade 9–12	122	21.4
	Diploma and above	31	5.4
Participants current job	Farmer	349	61.3
	House wife	111	19.5
	Employee	22	3.9
	Student	87	15.3
Traveling time to the nearby health facility	≥30 minutes	342	60.1
	< 30 minutes	227	39.9
Family history of hypertension	Yes	38	6.7
	No	531	93.3
Participant’s history of chronic illness	Yes	96	16.9
	No	473	83.1
Participant’s house hold wealthy index	Poor	227	39.9
	Medium	144	20
	Rich	228	40.1

### Participant’s behavior related characteristics

Of the total number of study participants, 62 (10.9%) had used tobacco products, including 38 (6.67%) smokers and 15 (2.63%) smokeless tobacco users, while nine (1.5%) of the study participants used both smoking and smokeless tobacco products before the time of data collection. About one-third, 201 (35.3%) of the study participants used alcohol products in their lifetime. More than half, 110 (54.7%), were current alcohol users. The majority of 187 (93%) of them consumed mainly locally produced alcohol. As for khat users, more than a quintile 123 (21.6%) of the study participants were Khat users (Table 3).

**Table 3:** Distribution of behavioral characteristics among adults living in rural area of Dano district, Oromia, Ethiopia, 2020 (n = 569).

Variables	Category	Frequency	Percentage
Ever used tobacco product	Yes	62	10.9
	No	507	89.1
Ever consumed alcohol	Yes	201	35.3
	No	368	64.7
Current alcohol users	Yes	110	19.3
	No	459	80.7
Favorite alcohol	Locally prepared	187	32.9
	Fabricated	14	2.5
Khat users	Yes	123	21.6
	No	446	78.4

### Participant’s Knowledge about the causes risk factors and prevention methods of hypertension

Most 556 (97.3%) of the study participants have heard at least about hypertension. Friends (44.5%) and family (33.9%) were the major sources of information in the study area.

The most common cause of hypertension reported was, increasing blood volume 404 (72.7%), stress 389 (70%), old age 292 (52.5%), and drug 206 (37.1%). The most common risk factor of hypertension reported was high fat intake 424(76.3%), being overweight 414 (74.5%), and high salt intake 359(64.6%). About,481(86.5%) reported that hypertension is preventable and the commonly reported prevention method was reducing fat intake 415(74.6%), reducing salt intake 402(72.3%), being physically active 373 (67.1%), reducing weight 334(60.1) and reducing smoking 219 (39.4%). Generally, nearly half 276 (48.5%) of the study participants had low knowledge about the cause, risk factor, and prevention methods of hypertension (Table 4).

**Table 4:** Distribution of participant’s knowledge of the cause, risk factors and prevention methods of hypertension among adults living in a rural area of Dano district, central Ethiopia, 2020 (n = 569).

Variables	Category	Frequency	Percentage
**Ever heard about hypertension**	Yes	556	97.7
	No	13	2.3
**Source of information**	Family	193	33.9
	Friend	253	44.5
	Social media	41	7.2
	Health care workers	69	12.1 2.1
**Composite Knowledge score**	**Good knowledge**	**293**	**51.5**
	**Low knowledge**	**276**	**48.5**

### Prevalence of undiagnosed hypertension among adults

**Fig 2:**
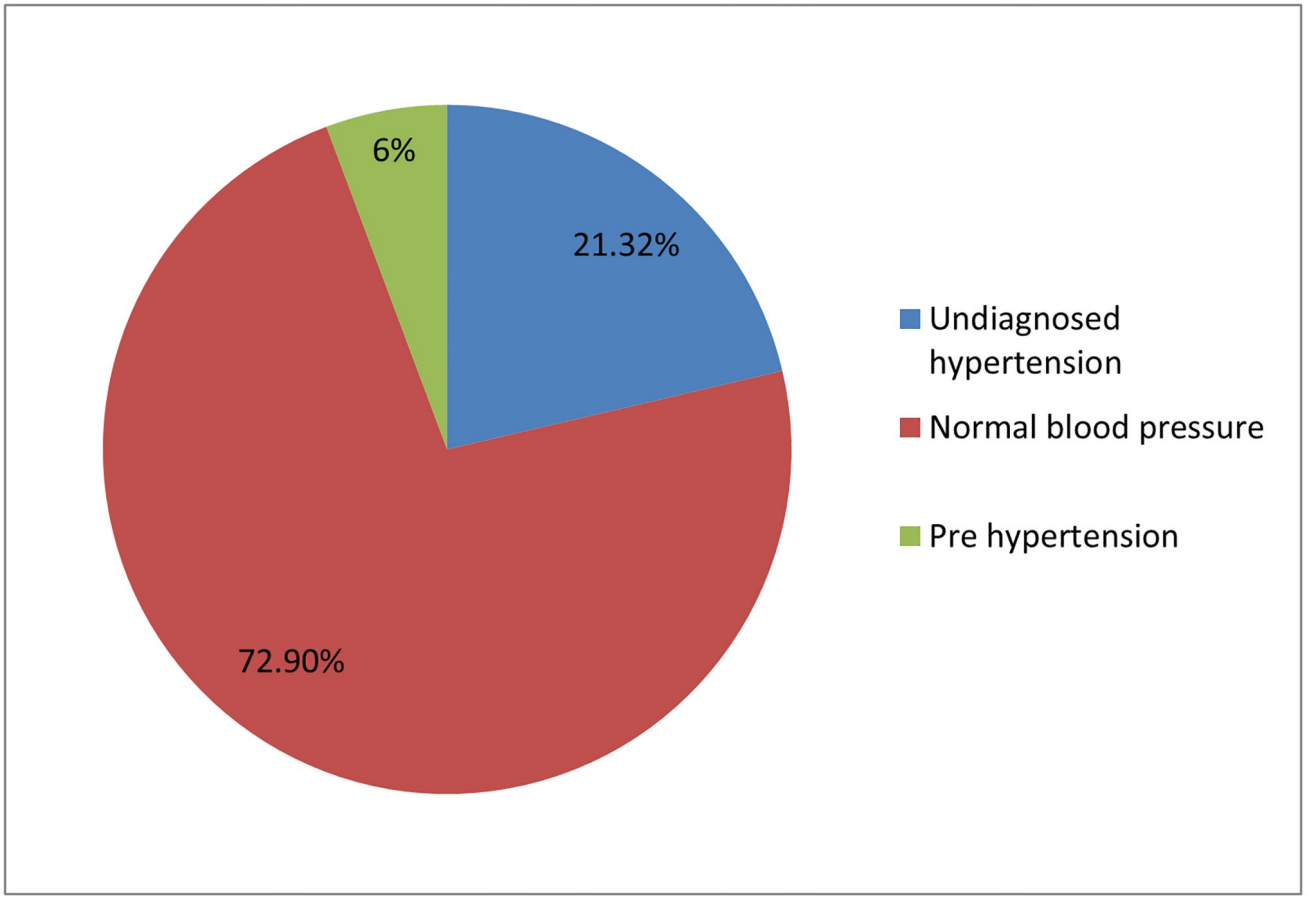
Magnitude of undiagnosed hypertension among adults living in rural of Dano District, Central Ethiopia, 2020.

The prevalence of undiagnosed hypertension in this study was 129(21.32%), CI: 19.95%, 25.8%). Out this, 19(14.72%) were systolic only hypertension and 110 (85.27%) were both systolic and diastolic hypertension and 6% of the participants are pre hypertensive (Fig 2).

### Factors associated with undiagnosed hypertension among adults

In bi-variable logistic regression, age of the participants, family history of hypertension, having a chronic illness, ever use of any tobacco products, currently smoking a cigarette, Khat users, participants’ knowledge about causes, risk factors, and prevention methods of hypertension, participant’s health-seeking behavior were associated with undiagnosed Hypertension among adults living in the rural area of Dano district.

In multi- variable regression analysis, participants’ wealth index, distance to the health facility, smokeless tobacco use, underweight, and adults’ knowledge of hypertension causes, risk factors, and prevention methods were found to be associated with undiagnosed Hypertension among adults living in the rural area of at P-value less than 0.05.

Those adults Living in a poor household (based on the household wealth index) were 3.1 times more likely to have undiagnosed hypertension [AOR: 3.156 (2.0, 6.3)] compared with the richest. Adults traveling a long distance to the health facility were 74.5% more likely to have their hypertension undiagnosed [AOR = 0.155 (0.11, 0.79)] than those traveling a short distance to the health facility. In this study, the odds of undiagnosed hypertension were 5.48 higher [AOR: 5.48 (3.34, 11.32)] in smokeless tobacco users than in those who did not smoke. This study reported that underweight adults had 2.2 more likely to have their hypertension undiagnosed compared with normal-weight adults [AOR = 2.2.1, 95%CI: (2.00, 4.22)]. Those adults who had low knowledge about the cause, risk factors, and prevention of hypertension had a 2.65-fold higher risk of undiagnosed hypertension [AOR = 2.65, 95%CI: (1.7, 4.95)] compared with their counterparts (Table 5).

**Table 5:** Factor associated with undiagnosed hypertension in multi-variable regression among adults living in rural area in Dano district, Central Ethiopia, 2020. (n = 569).

Associated factor	Category	Undiagnosed HTN		COR(95% CI)	AOR 95% CI	P-Value
		Yes (%)	No (%)			
Participant’s house hold wealthy index	Poor	80(62)	197(44.78)	3.27(1.4, 7.47)	3.156 (2.0, 6.3)*	0.0047
	Medium	35(27.14)	134 (30.45)	2.5(1.06, 6.0)	2.06 (0.75, 4.8)	0.174
	Rich	14(10.86)	109(24.78)	1	1	
Family history of HTN	Yes	10(12)	28(5.8)	2.24(1, 4.8)	3.1(1.23,7.78)	0.076
	No	73(88)	458(94.2)	1	1	
History of chronic disease	Yes	6(6.25)	90(93.75)	0.34(0.2, 0.81)	0.281(0.11,0.72)	0.058
	No	77(16.3)	396(83.7)	1	1	
Health seeking behavior	Low	69(83.1)	199(40.9)	7.1 (3.89, 12.9)	3.3 (1.6, 6.55)	0.058
	Good	14(4.65)	287(59.1)	1	1	
Knowledge status	Low	65(78.3)	211(76.4)	4.7(2.1, 8.1)	2.3 (1.2, 4.5)*	0.0024
	Good	18(21.7)	275(76.4)	1	1	
Distance to nearby health facility	≥30 minutes	44(53)	298(61.3)	.712(.446,1.137)	0.155 (.11.0.67)	0.001
	< 30 minutes	39(47)	188(38.7)	1		
Using smokeless tobacco	Yes	35	27	6.20(2.45,12.3)	5.7 (3.34,11.32) *	0.0015
	No	94	413	1	1	
Underweight	Yes	25	22	4.84(2.05,7.0)	5.56(3.45,7.89) *	0.001
	No	104	418	1		

NB: AOR = Adjusted odds ratio, COR = Crude odds ratio, CI = Confidence interval, HTN = Hypertension, * statistically significant.

## Discussion

The prevalence of undiagnosed hypertension in the study area was 21.32% (CI: 19.95%, 25.8%). In this study, participants wealth index, distance to the health facility, smokeless tobacco use, underweight, and adults’ knowledge of hypertension causes, risk factors, and prevention methods were identified as factors associated with undiagnosed hypertension in the study area.

The prevalence of undiagnosed hypertension in this study is comparable with the prevalence reported by the study done in, Nekemte town and Dire Dawa city [28,29]. and another study conducted in Ethiopia country wide (15.6%), Hawela-Tula sub-city(12.3%),Gulale,(13.25%) [19,30,31] and the present study finding is higher when compared to study done in USA(6.5%) and Gilgal-gibe field research (7.5%) [32,33]. The variation might be due to the difference in screening strategy the USA undertaking and the difference in the population studied whereas lower age cut-off point for the Gilgal gibe study.

This study indicated that those Adults who are living in poor households were 3 times more likely to have undiagnosed hypertension when compared to the richest. This might be due to the fact that, those participants living in poor household have less likely to visits the health care facility due to their socio-economic concern, which could decrease the probability of having been diagnosed for hypertension compared with rich participants. This finding is supported by the studies done in china [34]. This study found that, those Underweight adults were 5.56% more likely to have undiagnosed hypertension when compared to those who had not. This might be due to the misconception that hypertension is affect only the obese individual which might mislead the participants and health care provider to screen and participants to be screened for hypertension. This finding is in line with a study conducted in India [35]. This study revealed that those individuals leaving long distance from health facility were more likely to have undiagnosed hypertension compared to the counterpart. This finding is supported by the done in Bangladesh [36].

This study reviled that, those adults having low knowledge about the cause, risk factors and prevention method of hypertension were 2.3 times more likely to have undiagnosed hypertension when compared with an adult having good knowledge about the causes, risk factors and prevention methods of hypertension. This might be due to the reality that those adults having good knowledge about hypertension are more likely to seek health care and so that they can be diagnosed for hypertension. This finding is in line with the study done in Nigeria and Ethiopia [37,38]. This study showed that those adults having a family history of Hypertension were 3.1 more likely to have undiagnosed hypertension. This might be due to the fact that hypertension tends to run among families [39]. This finding is similar to the study done in Ethiopia [30].

## Conclusion and implications for practice

Undiagnosed hypertension is a considerable public health problem among adults living in the rural of Dano district. The study also demonstrated that almost one fifth of the study participants had undiagnosed hypertension and that living in a household with a low wealth index, being far from a health facility, being underweight, using smokeless tobacco products, and having little knowledge about hypertension were associated with higher odds of having undiagnosed hypertension, whereas, having other chronic illness was associated with reduced the odds of undiagnosed hypertension among adults living in the rural area.

## Implications for practice

Our main aim in this study was to assess the magnitude of undiagnosed hypertension and its associated factors in Adults residing in rural areas. We quantitate the magnitude and the possible associated factors of undiagnosed hypertension. Accordingly, the first major practical contribution of the present research is that it provides much-needed empirical data on the actual jobs of hypertension screening strategy based on the identified factors in this study. This information is important given that, there is a limitation of the study in the rural area of Ethiopia. The finding of this study will allow the NCD stakeholders, trainers, consultants, and others to design initiatives based on what has been identified as the risk factor of undiagnosed Hypertension. In this sense, we believe that our research is especially timely to meet the WHO strategy to tackle hypertension and related complications as one of the NCD global targets to decrease Non-communicable diseases, particularly in the rural community.

### Strength and limitation of the study

The study was unique, as it comprehensively examined the health factors associated with undiagnosed hypertension and provide cues for future hypertension prevention programs.

Despite these strengths, due to the nature of the study design (Cross-sectional study design), inferring the causality was not possible. Self-reported data (e.g., history of chronic disease &family history of HTN) might have recall bias. Fruit and vegetable consumption was measured based on the last 7 days’ experience, which could be too short to determine the status.

## Supporting information

S1 File (RAR)
